# The role of non-coding RNAs in the formation of long-term associative memory after single-trial learning in *Lymnaea*

**DOI:** 10.3389/fnbeh.2022.1005867

**Published:** 2022-10-14

**Authors:** György Kemenes, Paul R. Benjamin, Ildikó Kemenes

**Affiliations:** Sussex Neuroscience, School of Life Sciences, University of Sussex, Brighton, United Kingdom

**Keywords:** single-trial associative learning, long-term memory, non-coding RNA, CREB, NOS, *Lymnaea*

## Abstract

Investigations of the molecular mechanisms of long-term associative memory have revealed key roles for a number of highly evolutionarily conserved molecular pathways in a variety of different vertebrate and invertebrate model systems. One such system is the pond snail *Lymnaea stagnalis*, in which, like in other systems, the transcription factors CREB1 and CREB2 and the enzyme NOS play essential roles in the consolidation of long-term associative memory. More recently, epigenetic control mechanisms, such as DNA methylation, histone modifications, and control of gene expression by non-coding RNAs also have been found to play important roles in all model systems. In this minireview, we will focus on how, in *Lymnaea*, even a single episode of associative learning can activate CREB and NO dependent cascades due to the training-induced up- or downregulation of the expression levels of recently identified short and long non-coding RNAs.

## Introduction

For more than 20 years now the pond snail *Lymnaea stagnalis* has been providing highly valuable experimental models for analyses of the molecular mechanisms of associative memory. Using classical and operant conditioning paradigms, the mechanisms of the consolidation, maintenance, retrieval, forgetting, and reconsolidation of associative memory have been investigated successfully and these have been discussed in several recent reviews (e.g., Fodor et al., 2020; Kuroda and Abe, 2020; Rivi et al., 2020, 2021) and book chapters (e.g., Benjamin and Kemenes, 2017; Byrne et al., 2017; Benjamin et al., 2021). In *Lymnaea*, associative long-term memory (LTM) forms after multi-trial reward and aversive conditioning but notably, also after single-trial reward or aversive conditioning (Alexander et al., 1984; Kemenes et al., 2002; Martens et al., 2007; Sugai et al., 2007).

Molecular mechanisms of LTM formed after associative learning in *Lymnaea* involve the activation of evolutionarily highly conserved signaling pathways, such as NO/cGMP, cAMP/PKA, MAPK, GluR1, and NMDA receptors, CaMKII, insulin, transcriptional regulation of gene expression by CREB and C/EBP and the *de novo* synthesis of proteins (reviewed in Kemenes, 2015; Rivi et al., 2020). Epigenetic mechanisms also play important roles in the consolidation and enhancement of associative memory in *Lymnaea* (Lukowiak et al., 2014; Rothwell and Lukowiak, 2017; Korneev et al., 2018, 2021). The key roles these molecular pathways play in the formation of associative LTM in *Lymnaea* further confirm the generality of these highly conserved mechanisms, not only across phylogenetic groups but also across different types of learning (non-associative or associative, single- or multi-trial, aversive or reward, operant or classical).

Our main current interest is the molecular mechanisms underlying the consolidation of LTM after single-trial learning. Both everyday experience and numerous behavioral studies in animals and humans suggest the general importance of repetition for the formation of enduring memories after learning. However, in association with other stimuli, a single but highly salient event also can trigger LTM, a well-known example of which is “flashbulb” memory in humans. Although flashbulb memory has a specific definition (it is a detailed and vivid memory most people store on one or another occasion and retain for a lifetime, Brown and Kulik, 1977; Bartsch et al., 1995) and it has been studied most extensively in psychiatry (Sierra and Berrios, 1999), it shares a fundamental biological requirement with all other forms of single-trial induced associations: a single episode of learning must somehow gain immediate access to the complex molecular processing machinery known to be involved in the formation of LTM during multi-trial learning in all animal models of learning and memory (Kandel, 2001). The so far largely unanswered question of how this is achieved in the nervous system lies at the heart of understanding the conserved molecular mechanisms likely shared by all forms of learning resulting in LTM after just a single experience, from simple single-trial associative learning in animals to the formation of—often life-changing -complex flashbulb memories in humans.

A variety of different inhibitory constraints such as transcriptional repressors, non-coding RNAs, and histone deacetylases apply a continual brake on the molecular mechanisms associated with LTM, which is gradually relieved as repeat exposure suggests that this particular memory “is worth keeping.” However, flashbulb memory and all other forms of single-trial induced LTM (e.g., for a strongly aversive or highly rewarding stimulus) require the brake to be immediately released, allowing LTM formation. One of the hypotheses we have been testing in our recent studies is that non-coding RNA-induced downregulation of the expression of genes encoding specific inhibitory molecular constraints of memory consolidation is required for LTM to form after a single episode of learning and therefore learning-induced upregulation of such RNAs is required for its formation. Another hypothesis that has been investigated recently in the Sussex *Lymnaea* learning and memory laboratory is that some non-coding RNAs can repress the expression of genes encoding for specific enabling molecules of memory consolidation and therefore their downregulation is required for the formation of single-trial LTM.

To test these hypotheses, a combination of behavioral, pharmacological, and molecular methods was used in a top-down analysis of the role of specific recently identified non-coding RNAs (Korneev et al., 2018, 2021) in the rapid formation of single-trial LTM in the mollusk *Lymnaea stagnalis*. This experimental system provides a tractable model in which the most fundamental cellular and molecular mechanisms of LTM can be elucidated in the context of whole animal behavior as well as circuit and single neuronal activity (Kemenes, 2013). A unique advantage of this system is that LTM can be reliably induced by a single pairing of a neutral chemical conditional stimulus (0.004% amyl acetate, the CS) and a highly salient rewarding unconditional food stimulus (0.67% sucrose, the US), and therefore links between learning-related behavioral, molecular, and neuronal changes can be followed in a precisely timed manner. By contrast, classical conditioning using a mild tactile CS to the lips of *Lymnaea* paired with a slightly less concentrated (0.34%) but similarly, salient sucrose US (Kemenes et al., 1986) requires between five and 15 trials for LTM to form (Kemenes and Benjamin, 1989). Although the use of this multiple-trial protocol also provided important insights into the behavioral and neurophysiological mechanisms of appetitive learning (reviewed in Kemenes, 2013), only the single-trial protocol that has a sharply timed single phase of acquisition has been suitable for the investigation of learning-induced time-dependent molecular changes. Moreover, we can also exploit an *in vitro* version of the single-trial training protocol (Marra et al., 2013) where the formation of memory can be monitored “online,” recorded directly from key neurons in the feeding system.

The above experimental advantages make *Lymnaea* a uniquely powerful model for studying the cellular and molecular basis of the rapid formation of memory in a well-defined neuronal network. This research is very timely because although a number of major evolutionarily conserved molecular pathways that are necessary for LTM already have been identified in this and other invertebrate and vertebrate species (Kandel, 2001; Kemenes, 2013), it was not known in any system how a single episode of learning can downregulate the known inhibitory constraints on these molecular cascades to promote rapid memory consolidation. A thorough understanding of these key molecular pathways enabled the testing of the functional relationships of two of them, the CREB and NOS dependent pathways, respectively, with control mechanisms based on non-coding RNAs underlying single-trial associative LTM.

In this mini review we will focus on the role of two non-coding RNAs. The first one of these RNAs is a microRNA (miRNA), Lym-miR-137, which is involved in controlling the expression of the transcriptional repressor LymCREB2 (Korneev et al., 2018). The second RNA we will focus on is a long Natural Antisense Transcript (NAT), Lym-NOS1AS (Korneev et al., 2021). This antisense (AS) RNA is involved in repressing the expression of nitric oxide synthase (NOS), which produces NO that is required during the first 5 h post-training for LTM formation in *Lymnaea* (Kemenes et al., 2002).

## The miRNA Lym-miR-137 Targets CREB2 and Is Required for LTM After Single-Trial Classical Food-Reward Conditioning

The initial behavioral pharmacological analysis of the hypothesized role of miRNAs in single-trial induced LTM found that inhibition of the endoribonuclease Dicer by the injection of Poly-L-Lysine (PLL) 15 min after single-trial food-reward classical conditioning impaired LTM in *Lymnaea* (Korneev et al., 2018). This important observation demonstrated that the miRNA pathway is necessary for the consolidation of LTM in an early post-training time window. But notably, it also indicated that miRNAs may promote memory formation by silencing memory repressor genes rather than affecting memory enhancer ones. This was surprising because the loss of all Dicer-dependent miRNAs was shown to enhance rather than impair learning and memory in mice (Konopka et al., 2010), a finding seeming to show that the removal of mature miRNAs leads to the facilitation of translation of targeted synaptic genes playing key roles in synaptic plasticity.

In the next stage of the analysis in the *Lymnaea* model system, specific miRNAs with a potential role in LTM were identified using Next Generation Sequencing. The observed distribution of small non-coding RNAs in the cDNA libraries constructed as part of this analysis was similar to what was found in previous studies in *Aplysia* (Rajasethupathy et al., 2012).

The miRNA sequencing work discovered that a limited pool of miRNAs was differentially regulated by single-trial food-reward classical conditioning. An important finding was that most changes in the expression of these miRNAs occurred at 1 h after training, leading to the testable hypothesis that they play an important role during the early consolidation stage of long-term associative memory. The Korneev et al. (2018) study successfully demonstrated that Lym-miR-137, one of the miRNAs that showed transient upregulation 1 h after training, can form a stable duplex with mRNA encoding the CREB2 protein, a highly conserved transcription factor implicated in the repression of synaptic enhancement and memory in both vertebrates (Kida and Serita, 2014) and invertebrates, including *Drosophila* (Yin et al., 1994), *Aplysia* (Bartsch et al., 1995; Liu et al., 2011) and *Lymnaea* (Wagatsuma et al., 2006). Although there are several different ways by which CREB2 could interfere with transcription, according to the seminal Bartsch et al. (1995) *in vitro* study the most likely scenario is that CREB2 mediates repression by interacting directly with CREB1 (or another activator) to form an inactive heterodimer on the CRE region of a gene.

The Korneev et al. (2018) study provided several lines of evidence lending strong support to the notion that Lym-miR-137 promotes memory consolidation by targeting *Lym-CREB2* mRNA. These are as follows:

1. Lym-miR-137’s “seed” region is a 100% complementary to the putative target sequence in the *Lym-CREB2* mRNA and there is a high binding affinity between these two RNAs.
2. The training-induced transient increase of the level of Lym-miR-137 is followed by a transient decrease in the level of *Lym-CREB2*.
3. In the same group of experimental animals, pre-training treatment with a specific miR-137 inhibitor both upregulated the expression of *Lym-CREB2* mRNA and impaired LTM.
4. These two types of RNAs are co-expressed in the Cerebral Giant Cells (CGCs), an identified modulatory neuron type with an established role in LTM (Kemenes et al., 2006).

**Figure 1:**
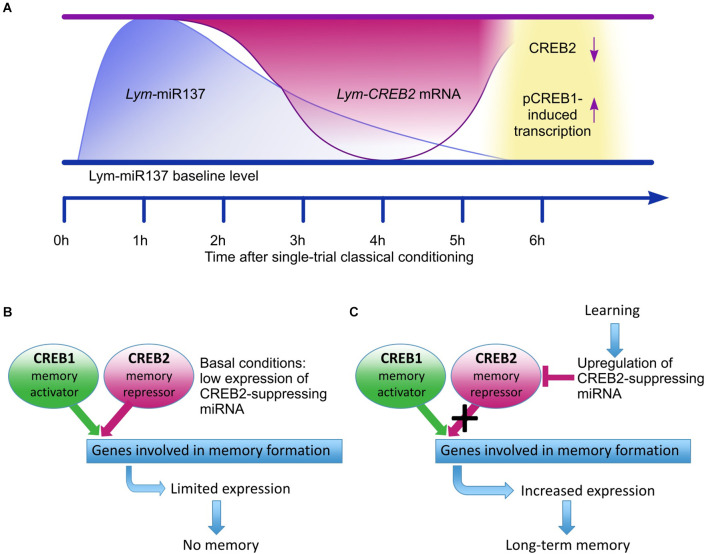
Adapted from Korneev et al. (2018). Schematic model for the proposed role of Lym-miR-137 in the regulation of CREB-dependent LTM formation in *Lymnaea*. **(A)** A schematic of the proposed temporal relationships between the learning-induced changes in Lym-miR-137 and *Lym-CREB2* mRNA levels and pCREB1-induced gene transcription. Lym-miR-137 levels were measured at 1 h and 6 h post-training, *Lym-CREB2* mRNA levels were measured at 4 h and 6 h post-training, pCREB1 levels were measured, and transcription dependence established at 6 h post-training (Korneev et al., 2018). **(B)** Under basal conditions, CREB1 and CREB2 compete for binding to the cAMP response elements (CRE) of target genes. Consequently, expression of CRE-dependent genes remains limited. **(C)** Single-trial training increases Lym-miR-137 expression resulting in down-regulation of CREB2. The repressive effect of CREB2 is decreased and CREB1 activates the expression of target CRE-dependent genes required for LTM formation.

The main conclusion from the Korneev et al. (2018) study is that in the learning and memory circuit of *Lymnaea* Lym-miR-137 plays an essential role by reducing the expression of *Lym-CREB2* mRNA and thus removing an important molecular “brake” of the CREB1-activated formation of LTM (Figure 1).

CREB1 is a highly conserved key transcriptional activator of learning-induced downstream molecular cascades underlying the consolidation of LTM in both invertebrates and vertebrates (Kandel, 2012; Kida and Serita, 2014). Although there is experimental evidence for learning-induced upregulation of CREB1 phosphorylation after single-trial appetitive conditioning in *Lymnaea* (Ribeiro et al., 2003), due to the lack of CREB-specific pharmacological inhibitors and availability of whole animal level genetic methods in this mollusk, we do not have direct evidence for LTM being dependent on CREB1. However, the pharmacological inhibition of each of the evolutionarily conserved key upstream activators of CREB1, such as PKA, MAPK, and CaMKII resulted in impaired behavioral single-trial induced LTM in *Lymnaea*, providing indirect evidence for the necessity of CREB1 for its consolidation. Moreover, using genetic constructs and manipulations, LTM and long-term synaptic plasticity induced by behavioral learning and its *in vitro* analogs in *Aplysia*, *Drosophila*, and mice have been shown to be dependent on CREB1 in identified neurons or specific brain circuits reviewed in Yin and Tully (1996), Kandel et al. (2014), and Kida and Serita (2014). Notably, similar to the original findings in identified sensory neurons of *Aplysia* (Dash et al., 1990), in *Lymnaea*, the injection of a CRE oligonucleotide into identified neurons (the CGCs in the case of *Lymnaea*), which express both the CREB1 protein and *Lym-CREB1* mRNA (Ribeiro et al., 2003; Sadamoto et al., 2004), inhibited long-lasting synaptic plasticity (Sadamoto et al., 2004).

In *Aplysia*, it also has been reported that downregulation of *CREB2* gene expression leads to increased long-term synaptic facilitation (Rajasethupathy et al., 2012). Furthermore, the CREB1/CREB2 ratio can determine the consolidation of LTM in this species (Liu et al., 2011). These findings suggested that when the CREB1/CREB2 ratio increases, the transcriptional machinery necessary for LTM is more readily activated. Moreover, other studies in *Lymnaea* have shown that a *CREB1*-specific siRNA injected into the CGCsblocks, while a *CREB2*-specific siRNA augments the enhancement of excitatory post-synaptic potentials between the CGCs and B1 motoneurons, a readout of learning-induced presynaptic facilitation (Wagatsuma et al., 2006). These observations demonstrate that prevention of the synthesis of new CREB2 molecules by interfering with the *CREB2* mRNA results in reducing their repressing effect on CREB1. Thus, the Korneev et al. (2018) study revealed a novel endogenous miRNA-dependent mechanism resulting in the down-regulation of CREB2, which facilitates LTM formation after single-trial conditioning.

Although the Korneev et al. (2018) study conclusively showed that inhibiting Dicer-mediated miRNA biogenesis with PLL impairs LTM formation, as measured by the feeding response to the amyl acetate CS 24 h after training, a recent study demonstrated that inhibiting miRNAs also affects *Lymnaea’s* sense of taste. Kagan et al. (2022) replicated the effect of PLL injection on LTM after the same single-trial appetitive classical conditioning procedure that was used in the Korneev et al. (2018) study but they also found that inhibition of miRNA biogenesis resulted in reduced feeding responses to food stimuli with a previously high hedonic value, including sucrose (the hedonic value is a measure of how pleasurable a sensation is). According to Kagan et al. (2022), this finding seemed to suggest that PLL causes anhedonia rather than impaired LTM consolidation after single-trial conditioning but later in their discussion they concluded that PLL treatment may both prevent LTM formation and reduce the hedonic value of food stimuli in *Lymnaea*. The Korneev et al. (2018) study found a significant reduction in the feeding response to sucrose at 2 h but not 24 h after PLL injection, so the reduced response to amyl acetate 24 h after training was unlikely to be due to a general blunting of the sense of taste of the PLL-treated trained animals. It is unclear why the 24-h post-PLL feeding scores are different between the two studies, but Kagan et al. (2022) themselves noted that the reduced feeding response 24 h after PLL injection could be a result of different environmental conditions of rearing compared to those of the Korneev et al. (2018) study (Rothwell and Lukowiak, 2019). Finally, the Korneev et al. (2018) study also used a specific Lym-miR-137 inhibitor *in vivo*, which resulted in impaired 24 h LTM. It would be interesting to test whether or not inhibiting Lym-miR-137 alone reduces the hedonic value of sucrose under the conditions of the Kagan et al. (2022) study where inhibiting miRNA biogenesis, in general, was observed to have this effect.

miR-137 also has been implicated in memory formation in mammals but with some controversy concerning its role. One study concluded that miR-137 promotes the formation of spatial memory (Huang et al., 2014), while a different study demonstrated that overexpression of miR-137 impairs synaptic plasticity and contextual fear conditioning (Siegert et al., 2015). Also taking into account the findings of the Korneev et al. (2018) study, it is reasonable to conclude that the apparent differences in the role of miR-137 in the formation of memory can be explained by differences in the types of learning investigated. Depending on the CS-US associations used to induce LTM, miR-137 may interact with different target RNAs and thus may initiate different downstream events. It is well-established in both vertebrate and invertebrate model systems that the same upstream molecular components can play different roles in different types of learning and the above-discussed findings regarding miR-137’s different roles in three distinct types of associative memories fit well into this general framework.

## The Long Natural Antisense Transcript *Lym-NOS1AS* Targets NOS and Represses LTM After Single-Trial Classical Food-Reward Conditioning

Two types of NOS-encoding mRNAs are expressed in the *Lymnaea* brain: *Lym-nNOS1* and *Lym-nNOS2* (Korneev et al., 2005). Notably, the expression of *Lym-nNOS1* is temporally differentially regulated by single-trial food-reward conditioning, but the level of *Lym-nNOS2* expression remains stable during the same post-training time period (Korneev et al., 2005). The antisense (AS)RNA of *Lym-nNOS1, Lym-NOS1AS*, is a long natural antisense transcript (NAT) identified in 2021 by Korneev et al. and is complementary exclusively to the *Lym-nNOS1* mRNA. This finding, in combination with its temporally different post-training expression levels (Korneev et al., 2021) led to the formulation of the hypothesis that the regulation of the expression of *Lym-nNOS1*, it is an important component of the LTM consolidation pathway.

To test this hypothesis, Korneev et al. (2021) first utilized one of the principal advantages of the *Lymnaea* model system, which is the feasibility to investigate molecular processes at the level of single identified neurons. Using RT-PCR they found that the CGCs co-express both the *Lym-nNOS1*mRNA and the *Lym-NOS1AS* long non-coding RNA, which suggested that there was an interaction between these NOS-related sense and antisense RNAs in the same neuron, with a potential role in synaptic plasticity and memory formation.

**Figure 2:**
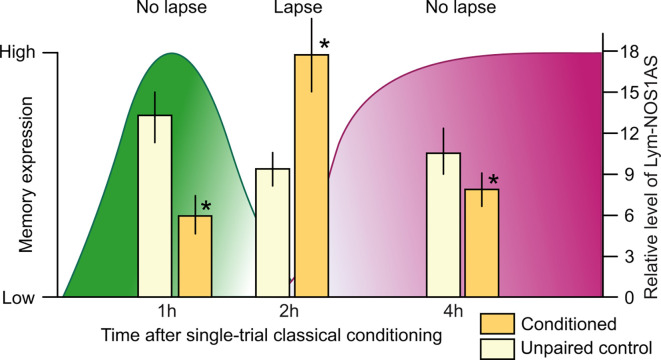
Correlation between memory lapse/non-lapse periods and the level of *Lym-NOS1AS* expression after single-trial classical food-reward conditioning in *Lymnaea*. The levels of memory expression are indicated on the left axis, based on data from Marra et al. (2013). The levels of *Lym-NOS1AS* expression are indicated on the right axis, based on data from Korneev et al. (2021). *Lym-NOS1AS* expression levels are significantly lower at the 1 h and 4 h non-lapse time points in the cerebral ganglia from the trained group of animals (yellow bars) compared to controls (white bars). By contrast, at the 2 h lapse time point, *Lym-NOS1AS* expression level is significantly higher in the cerebral ganglia of trained vs. control animals. Asterisks indicate significant differences (*p* < 0.05) between the conditioned and unpaired data at the same time point (*n* = 20 animals per group, unpaired two-tailed *t*-tests with Welch’s correction).

To further investigate the role of *Lym-NOS1AS*, the Korneev et al. (2021) study investigated whether it is also regulated at the systems level by single-trial food-reward classical conditioning. In a large-scale quantitative experiment, the expression levels of *Lym-NOS1AS* were measured in the buccal and cerebral ganglia the “learning” ganglia that contain the neural circuits necessary for LTM formation (Straub et al., 2004, 2006) at different time points after conditioning. This analysis revealed that *Lym-NOS1AS* expression in the learning ganglia is differentially regulated by training in a time-dependent manner: it is either downregulated or upregulated in the cerebral ganglia at specific time points (Figure 2) but remains stable in its expression in the buccal ganglia (Korneev et al., 2021). Furthermore, in the cerebral ganglia, *Lym-NOS1AS* expression is transiently decreased at 1 h and 4 h but transiently increased at 2 h after conditioning (Figure 2). To conclude, the learning-induced time-dependent changes in *Lym-NOS1AS* expression are restricted to the cerebral ganglia, where most of the NO-dependent information processing takes place after single-trial learning (Korneev et al., 2005). Furthermore, these changes occur at specific times during a critical phase of memory consolidation (Marra et al., 2013) when NO is essential for LTM (Kemenes et al., 2002).

Memory consolidation in *Lymnaea*, just like in other organisms including humans, goes through different phases, with intervals when the memory temporarily becomes weak and vulnerable to interference (Marra et al., 2013). One such “lapse” period occurs at 2 h post-training. In contrast, the 1 h and 4 h post-training time points are “non-lapse” periods, when the memory is fully expressed in response to the CS. The Korneev et al. (2021) study revealed a correlation between the lapse/non-lapse periods and the level of *Lym-NOS1AS* expression: the 1 h and 4 h non-lapse periods coincide with the downregulation of this NAT, whereas the 2 h lapse period coincides with its upregulation (Figure 2). This suggests that the disengagement of the *Lym-NOS1AS* “NO brake” at 1 h and 4 h post-training enables NO synthesis, which explains the robustness of the NO-dependent memory trace at these time periods. And the opposite is also true: the engagement of the brake at 2 h post-training suppresses NO synthesis and therefore may account for the observed temporary interruption of the NO-dependent phase of memory consolidation.

On balance, it seems that compared to CREB2, *Lym-NOS1AS* represents another important type of memory repressor, long antisense RNAs interfering with the mRNAs of key molecular players of memory consolidation and therefore acting as memory constraints. The correlation between the behavioral and RT-PCR findings established by the Korneev et al. (2021) study and shown in Figure 2 suggests that for LTM to form after a single trial, this memory constraint must be absent or reduced during the NO-dependent phase of memory consolidation. However, whether the spatially and temporally targeted removal of the brake provided by the *Lym-NOS1AS*at a memory lapse point at 2 h post-training is essential for or simply facilitates LTM formation has yet to be established.

## Discussion

The studies that have been reviewed here revealed two different novel mechanisms by which non-coding RNAs can interfere with well-known molecular processes of memory consolidation after single-trial classical food-reward conditioning. The miRNA Lym-miR-137 acts by downregulating the expression of a known repressor of memory consolidation, CREB2, whereas the long natural antisense transcript *Lym-NOS1AS*, downregulates the expression of NOS, a key molecule of the molecular cascades enabling memory consolidation. Levels of both of these non-coding RNAs are significantly affected by single-trial food-reward training but in a fundamentally different way, with the expression of Lym-miR-137 upregulated whereas the expression of *Lym-NOS1AS* downregulated 1 h after training. However, both of these opposite changes have the effect of weakening an inhibitory molecular constraint on the formation of LTM. A key finding from the experiments on *Lym-NOS1AS* was that changes in the level of its expression could be correlated to memory lapse and non-lapse periods that occur during the early consolidation period (up to 4 h) post-training.

Whether in *Lymnaea* the CREB and NO dependent cascades that are affected by the two different non-coding RNAs reviewed here are parts of two independent pathways (the former activated by PKA while the latter activated by PKG), or both are parts of the same pathway remains to be elucidated. If it is the latter, one possible link is provided by the observed early dependence of LTM on both cAMP/PKA and NO/cGMP after single-trial food-reward classical conditioning (Kemenes et al., 2002, 2006; Michel et al., 2008). In a recent article by Farruggella et al. (2019), it was suggested that in *Aplysia* NO might work synergistically with 5-HT to amplify the activity of PKA *via* cAMP activation, the main driver of CREB-mediated transcriptional activation and a similar interaction might occur in *Lymnaea*. Although this is a very plausible mechanism, as the same authors also discuss in their paper, in the honeybee *Apis mellifera* (Müller, 2000) and the cricket *Gryllusbimaculatus* (Matsumoto and Mizunami, 2006), where, similar to all other model systems, the activation of PKA is required for long-term associative memory formation, it is triggered by a NO/cGMP pathway. An early seminal study in mice showed that NO contributes to late-phase LTP by stimulating sGC- and cGMP-dependent protein kinase (PKG), which acts in parallel with cAMP-dependent protein kinase (PKA) to increase the phosphorylation of CREB (Lu et al., 1999). Taken together it is therefore conceivable that some key molecular mechanisms linking NO to CREB-dependent processes underlying LTM formation are shared by these two pathways in both invertebrate and vertebrate models of long-term memory.

## Data Availability Statement

The behavioral data generated by the original contributions presented in the study are publicly available. This data can be found here: Research data from the behavioral experiments described in the reviewed paper Korneev et al. (2018): ‘A CREB2-targeting microRNA is required for long-term memory after single-trial learning’. University of Sussex. Dataset. https://doi.org/10.25377/sussex.5809716.v1. Research data from the behavioral experiments described in the reviewed paper Korneev et al. (2021): ‘Time dependent differential regulation of a novel long non-coding natural antisense RNA during long-term memory formation’. University of Sussex. Dataset. https://doi.org/10.25377/sussex.13664204.v1. All real-time RT-PCR data generated by the original contributions presented in the study are included in the above published articles, which are publicly available under DOI: 10.1038/s41598-018-22278-w and DOI: 10.1038/s41598-021-83190-4, respectively.

## Author Contributions

GK, PB, and IK discussed and agreed on the plan for the mini review. GK wrote the first draft of the manuscript. PB and IK both contributed to the submitted version. IK created the figures. All authors contributed to the article and approved the submitted version.

## Funding

The experimental work that generated the data for the non-coding RNA studies reviewed here was supported by the Biotechnology and Biology Research Council grant BBSRC/BB/P00766X/1 to IK, GK, and PB.

## Conflict of Interest

The authors declare that the research was conducted in the absence of any commercial or financial relationships that could be construed as a potential conflict of interest.

## Publisher’s Note

All claims expressed in this article are solely those of the authors and do not necessarily represent those of their affiliated organizations, or those of the publisher, the editors and the reviewers. Any product that may be evaluated in this article, or claim that may be made by its manufacturer, is not guaranteed or endorsed by the publisher.
